# Mechanization-driven farmland consolidation and farm household labor allocation: Evidence from grain producers in Shandong, China

**DOI:** 10.1371/journal.pone.0340297

**Published:** 2026-02-06

**Authors:** Yang Liu, Pengsong Ding, Xiubo Xia, Shuping Li, Qingpeng Sun, Tianjing Yang, Yufei Bu, Huanchun Zhang

**Affiliations:** 1 Yantai Agricultural Science Research Institute, Yantai, Shandong, China; 2 Yantai Agricultural Technology Extension Center, Yantai, Shandong, China; Federal University of Agriculture Abeokuta, NIGERIA

## Abstract

Mechanization-driven farmland consolidation has become a key component of China’s efforts to raise grain productivity and optimize rural labor allocation. We used a survey of 630 grain-producing households in Shandong Province, and combined a Tobit model with propensity-score matching to identify the causal effects of consolidation on farm-household labor decisions. Consolidation reduced on-farm labor input by 8.4 percentage points through labor-saving technological substitution, yet the magnitude differed sharply between two mechanization pathways. Where households purchased their own machinery, on-farm labor rose by 5.3 percentage points, consistent with specialization incentives. By contrast, the use of custom mechanization services lowered on-farm labor by 7.5 percentage points. Labor-saving effects were strongest among ageing households, smallholders and farmers in hilly areas, suggesting enhanced overall efficiency in constrained settings. Policy implications include expanding service markets, coupling consolidation with vocational training for off-farm employment, and establishing a long-run monitoring framework to ensure sustainable transformation. However, this study relies on cross-sectional data, which limits its ability to capture dynamic change processes. Future research could conduct longitudinal tracking studies to evaluate the sustained effects and sustainability of policies.

## Introduction

In recent years, food security has become a national strategic priority in China, with significant emphasis placed on agricultural infrastructure development [1,2]. Mechanization-driven farmland consolidation has emerged as a critical measure to enhance agricultural productivity and ensure stable grain production [3,4]. Since the implementation of the 14th Five-Year Plan (2021–2025), China has prioritized mechanization-driven farmland consolidation, allocating substantial policy support and financial resources to comprehensive land leveling, irrigation and drainage systems, field roads, and digital infrastructure [5]. These efforts have improved farmland quality and agricultural production conditions. As one of China’s major grain-producing provinces, Shandong has actively responded to national policies by promoting large-scale mechanization-driven farmland consolidation. This initiative aims to boost grain yields, optimize regional agricultural structures, and reinforce Shandong’s strategic role in the national grain production landscape. However, despite the growing sophistication of mechanization-driven farmland infrastructure, systematic analyses of its micro-level impacts remain limited, particularly regarding the allocation of production factors and productivity among grain-producing households [6,7].

Existing studies have predominantly focused on macro-level productivity effects, with scant attention to micro-level behavioral adjustments in labor allocation. Nevertheless, three critical gaps persist in research on policy-induced changes in farmer behavior, resource allocation adjustments, and their deeper implications for production efficiency [8–10]. First, empirical research on how farmers reallocate labor [11], land [12], capital [13], and technology [14] under mechanization-driven farmland consolidation, how such reallocation enhances agricultural output efficiency remains scarce [15]. Second, most studies rely on county- or township-level data [16], lacking robust micro-level foundations [17], detailed pathway analyses, or sufficient consideration of regional heterogeneity [18,19]. Consequently, there is an urgent need for finer-grained household panel data to accurately quantify the micro-level effects of mechanization-driven farmland consolidation policies.

This study systematically analyzes how mechanization-driven farmland consolidation influences agricultural labor allocation and productivity, using survey data in Shandong Province. We develop a comprehensive analytical framework that distinguishes between two mechanization pathways: self-owned machinery and outsourced mechanization services. Robust empirical methods are employed to address endogeneity concerns. Empirical studies linking household-level reallocation of labor, land, capital, and technology to efficiency gains under mechanization-driven consolidation are still limited. Unlike previous macro-level research, this study aims to address the following core questions: (1) How does mechanization-driven farmland consolidation affect farm household labor allocation? (2) Are there differences in the impacts of different mechanization pathways on labor allocation? (3) Does this impact exhibit heterogeneity among farmer groups with different characteristics? Through the exploration of these questions, this study will provide empirical evidence and policy insights for advancing China’s agricultural modernization and optimizing rural labor allocation.

## Materials and methods

### Study area

**Fig 1:**
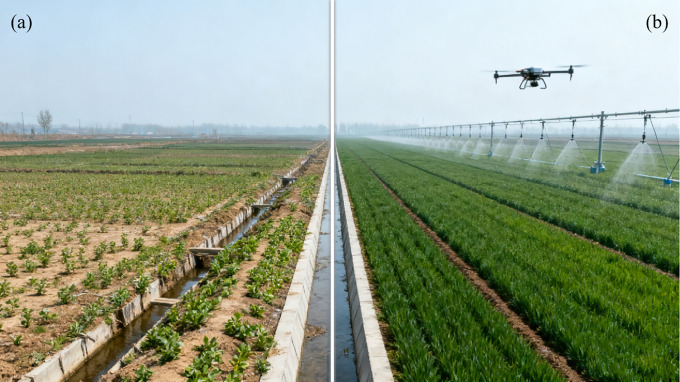
Comparison of mechanization-driven farmland and non-mechanization driven farmland scenarios. (a) Non-mechanisation-driven farmland consolidation; (b) Mechanisation-driven farmland consolidation.

Shandong Province, located in the eastern coastal region of China (34°22.9’–38°24.0′ N, 114°47.5′–122°42.3′ E), is a major grain-producing province. In 2024, its total grain output reached 57.102 million tons, accounting for 8.1% of the national total. The total arable land resources amount to 83.38 million ha, with a grain self-sufficiency rate consistently above 100% [20]. Shandong Province has cumulatively constructed 50.98 million ha of mechanization-driven farmland (accounting for 7.6% of the nation’s over 67 million ha of it) [21,22], with significant differences in topography and planting structure (Fig 1). The average age of the agricultural labor force was 55.6 years, with those over 65 years old accounting for 28.4% of the workforce. The land fragmentation index (average number of plots per household: 5.2) is higher than the national average (3.8 plots), and the mechanization rate is 86.5% (national average: 71.3%) [23], The average age of the agricultural labor force is 55.6 years, with those over 65 years old accounting for 28.4% of the labor force. Which highlights a paradox in Shandong Province: a high mechanization rate coexisting with significant labor constraints. This makes it an ideal case study for analyzing factor reconfiguration effects.

### Theoretical analysis and research hypothesis

#### The direct effect of mechanization-driven farmland consolidation on farm household labor allocation.

Farm household labor allocation is fundamentally influenced by family resource endowments and the comparative advantages of household members. As a core livelihood asset, farmland characteristics significantly shape labor decisions [24]. Mechanization-driven farmland consolidation, achieved through comprehensive infrastructure improvements such as land leveling, enhanced irrigation systems, and the construction of farm roads [25]. Affects agricultural labor allocation through two primary channels:

First, by reducing plot fragmentation [26], minimizing topographic undulations [27], and improving field accessibility [28] and irrigation facilities [29], mechanization-driven farmland consolidation significantly enhances production conditions. This reduces the difficulty and intensity of agricultural operations, thereby saving labor traditionally required for tasks such as inter-plot transfers. The labor-saving effect is most evident in reduced time spent on field preparation, water management, and land maintenance activities, aligning with the induced innovation theory [30]. This theory posits that mechanization adoption accelerates when the relative cost of labor rises.

Second, by improving land quality [31], soil fertility [32], and overall productivity [33] , mechanization-driven farmland consolidation may stimulate farmers’ enthusiasm for agricultural production, particularly among large-scale and specialized farming entities. This productivity-enhancing effect could encourage increased labor input into agriculture, as improved land quality and infrastructure raise the expected returns of agricultural activities. However, in China’s current agricultural landscape, dominated by small-scale and fragmented operations, the labor-saving effect often outweighs the productivity-enhancing effect [34]. This is because smallholders face higher opportunity costs for labor and prioritize non-agricultural employment opportunities for household members. Based on these mechanisms, we propose:

Hypothesis 1: Mechanization-driven farmland consolidation reduces agricultural labor input by improving field conditions and promoting mechanization adoption, thereby releasing labor for non-agricultural employment opportunities.

#### Divergent mechanization pathways and their impacts on labor allocation.

According to the theory of induced technological change, farmers substitute machinery for scarce labor when factor scarcities shift [35]. Mechanization-driven farmland consolidation optimizes conditions for mechanized operations and influences labor allocation through two distinct pathways, each with different theoretical implications.

When farmers invest in purchasing agricultural machinery, they typically commit to agricultural specialization as a long-term strategy [30]. According to the theory of induced technological change, farmers substitute machinery for scarce labor when factor scarcities shift [36]. This capital-deepening process strengthens agricultural production as farmers seek to maximize returns on machinery investments through higher utilization rates. Owned machinery provides operational flexibility but often requires supplementary labor for operation and management, shifting labor from manual tasks to machinery operation. Thus, for farmers choosing this pathway, mechanization-driven farmland consolidation may increase agricultural labor input as they allocate more resources to specialized production.

In contrast, farmers purchasing mechanized services adopt a labor-outsourcing strategy. By acquiring these services, farmers effectively outsource labor-intensive tasks to specialized providers, directly reducing their own labor demand [37]. Without the fixed costs of machinery ownership, farmers can flexibly allocate labor to non-agricultural activities when opportunities arise [38]. This pathway represents a risk-management strategy, particularly valuable for smallholders, enabling access to advanced technology without substantial capital investment while reallocating labor to more profitable opportunities.

**Fig 2:**
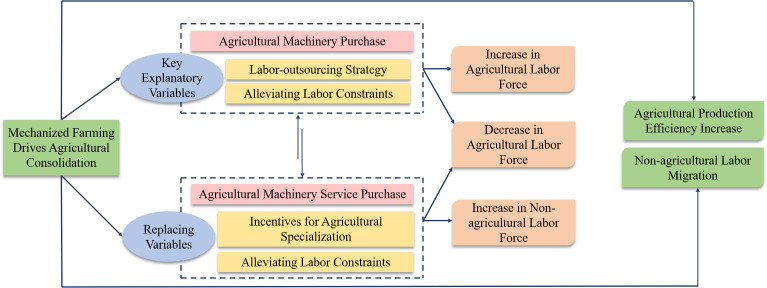
Theoretical Framework for Analyzing the Impact of Mechanization-driven Farmland Consolidation on Labor Allocation.

The divergent impacts of these pathways introduce a critical theoretical nuance in understanding how mechanization-driven farmland consolidation affects labor allocation. While both pathways benefit from improved field conditions, they represent distinct strategies: the ownership pathway typically increases agricultural labor through specialization, whereas the service-purchasing pathway reduces it through outsourcing and reallocation. The framework for the impact of mechanization-driven farmland consolidation on labor allocation is illustrated in Fig 2. Based on this analysis, we propose:

Hypothesis 2: Mechanization-driven farmland consolidation influences farm household labor allocation by accelerating the “machinery-labor” substitution process, but the effects vary by mechanization pathway. Specifically, it increases agricultural labor input by promoting machinery ownership and agricultural specialization, while reducing agricultural labor input by facilitating mechanized service purchases and alleviating labor constraints.

## Research methods

### Variable selection

(1) Dependent variable: Farm household labor allocation. Use the proportion of agricultural labor and non-agricultural labor to measure the labor force allocation status of farm households.
(2) Core Explanatory Variable: Represented by whether the household has constructed mechanization-driven farmland. If the household has constructed such farmland, it is assigned a value of 1; otherwise, it is assigned a value of 0.
(3) Key explanatory variables: agricultural machinery purchase, agricultural machinery service purchase. Among them, agricultural machinery purchase is measured by whether farmers have purchased agricultural machinery (such as rotary tillers, seeders, harvesters, tractors, and other agricultural machinery). If agricultural machinery is purchased, it is assigned a value of 1; otherwise, it is assigned a value of 0. Agricultural machinery service purchase is measured by whether farmers have purchased agricultural machinery services (such as machine tillage services, machine planting services, pest control services, and machine harvesting services). If agricultural machinery services are purchased, it is assigned a value of 1; otherwise, it is assigned a value of 0.

We include both “machinery purchase” and “mechanization service purchase” as key explanatory variables because they represent two primary pathways to mechanization and are expected to exert differentiated effects on the direction of labor input. The former is often accompanied by capital deepening and a tendency toward specialized operations, which may increase time spent on labor organization and management in agricultural activities and, under certain conditions, raise agricultural labor input. The latter substitutes for household labor by outsourcing labor-intensive tasks, thereby reducing farmers’ agricultural labor input and freeing up elasticity for off-farm employment. This variable design aligns with the study’s theoretical framework of the “machine–labor substitution” mechanism and enables identification of the heterogeneous effects of different mechanization modalities on labor allocation. Meanwhile, service purchases also reflect farmers’ preferences for flexibility and risk management under constraints related to liquidity, farm size, and plot fragmentation.

(4) Control Variables: Considering that household labor allocation is influenced by numerous factors, to ensure the validity of the estimation results, we select characteristics of agricultural production decision-makers, household characteristics, and village characteristics as control variables. These include the agricultural production decision-makers’ gender, age, years of education, health status, and whether they have received agricultural technology training; household characteristics include household size, degree of aging, agricultural land management scale, number of plots, agricultural land quality, total household income, proportion of agricultural income, experience with farmland consolidation, and agricultural production subsidies; village characteristics include the distance from the village committee to the township government, whether the village has secondary or tertiary industries, per capita agricultural land area in the village, and whether the village is located in a plain area. In addition, regional dummy variables are included to avoid the impact of unobservable variables that vary with regions on the estimation results. The specific variable settings and meanings are shown in Table 1.

**Table 1:** Variable Definitions and Descriptive Statistics (N = 630).

Variable Name	Variable Meaning	Mean (%)	SD (%)
**Dependent Variable**			
**Agricultural Labor Allocation Ratio**	Number of agricultural labors/ Total household labors force	0.509	0.233
**Non-agricultural Labor Allocation Ratio**	Non-farm workforce/Household workforce	0.365	0.412
**Agricultural Working Hours**	Total monthly labor hours that farmers spend on agricultural production	8.919	5.745
**Non-farm Labor Hours**	Total monthly labor hours spent by farm households on non-agricultural employment	10.851	11.612
**Core Explanatory Variable**			
**Mechanization-driven Farmland Consolidation**	Whether the farmer has developed mechanization-driven farmland: Yes = 1; No = 0	0.402	0.395
**Key Explanatory Variable**			
**Agricultural Machinery Purchase**	Whether the household has purchased agricultural machinery: Yes = 1; No = 0	0.312	0.462
**Purchase of Agricultural Machinery Services**	Whether the household purchases agricultural machinery operation services: Yes = 1; No = 0	0.784	0.412
**Decision-maker Characteristics**			
**Gender**	Gender of agricultural production decision-maker: Male = 1; Female = 0	0.812	0.365
**Age**	Age of agricultural production decision makers (years)	51.948	8.948
**Educational Background**	Years of schooling of agricultural production decision-makers (years)	7.626	2.568
**Health Status**	Health status of agricultural production decision-makers: Very unhealthy = 1; Somewhat unhealthy = 2; Average = 3; Somewhat healthy = 4; Very healthy = 5	4.021	0.956
**Agricultural Technology Training**	Has the agricultural production decision-maker received agricultural technology training: Yes = 1; No = 0	0.423	0.365
**Family Characteristics**			
**Household Size**	Number of farm household members (person)	3.147	1.623
**Aging Degree**	Proportion of population aged 65 and over in total household population	0.201	0.312
**Farmland Operation Scale**	Household farm land operating area (ha); Log-transformed	0.201	0.956
**Number of Plots**	Number of family farm land plots (units)	4.926	5.632
**Farmland Quality**	Household farmland quality: Very poor = 1; Poor = 2; Average = 3; Good = 4; Very good = 5	4.012	1.026
**Household Total Income**	Total household income total household income (RMB); Logarithmic transformation applied	9.658	0.985
**Proportion of Agricultural Income**	Proportion of agricultural income in total household income of farming families	0.298	0.412
**Farmland Consolidation Experience**	Apart from mechanization-driven farmland consolidation, has the farmer ever undergone farmland consolidation? Yes = 1; No = 0	0.316	0.364
**Agricultural Production Subsidy**	A mount of agricultural subsidies received by farmers (RMB); natural logarithm applied	4.265	2.365
**Village Features**			
**Distance From the Village to the Township Government Office**	Distance from village committee to township (km)	3.297	4.021
**Village Traffic Conditions**	Is the village adjacent to a national or provincial highway, or a county or township road: Yes = 1; No = 0	1.120	0.203
**Does the village have secondary and tertiary industries?**	Yes = 1; No = 0	0.562	0.562
**Per Capita Farmland Area in the Village**	Per capita agricultural land area in the village (ha/person)	0.269	1.983
**Is the Village Located on a Plain?**	Yes = 1; No = 0	0.502	0.515

Note: The degree of aging refers to the proportion of household members aged 65 and above within the total household population, reflecting the “aging” characteristics of the rural labor force. The number of plots indicates the actual number of farmland parcels managed by each household, used to measure the fragmentation of farmland.

These control variables are employed to mitigate omitted-variable bias and enhance identification strength. The decision-maker’s age and health status affect physical capacity and opportunity costs, thereby influencing labor input; educational attainment and agricultural-technology training shape technology adoption and preferences for outsourcing services; the number of plots and land quality capture fragmentation and production conditions, exerting direct effects on the feasibility of mechanization and the demand for labor; income and the share of agricultural income reflect household comparative returns and motivations for labor reallocation; and village-level location and industrial structure are linked to off-farm opportunities and accessibility. Taken together, these controls complement the economic meanings of the core explanatory variables and help purify the estimated relationship between mechanized high-standard farmland and labor input.

### Model setup

(1) Tobit model. The Tobit model was employed to empirical analysis of the impact of mechanization-driven farmland consolidation on farm household labor allocation. The specific model setup is as follows:

$$Labor\_farm_{i}=\alpha_{0}+\alpha_{1}High\_sd_{i}+\alpha_{2}Control_{i}+\varepsilon_{i}$$ (1)

$$Labor\_nofarm_{i}=\beta_{0}+\beta_{1}High\_sd_{i}+\beta_{2}Control_{i}+\varepsilon_{i}$$ (2)

Where Labor_farm_i_ and nofarm_i_ represent the proportion of agricultural labor force and non-agricultural labor force of the i-th farmer, respectively, High_sd_i_ represents the mechanization-driven farmland consolidation status of the i-th farmer (1 if mechanization-driven farmland is constructed, 0 otherwise); Control_i_ are control variables, α and β are parameters to be estimated, and is a random disturbance term.

(2) Ordinary Least Squares (OLS) Regression. In addition to using the relative indicators of agricultural labor force share and non-agricultural labor force share, we employed absolute indicators of total household agricultural labor time (in months) and total non-agricultural labor time (in months) to characterize farm household labor allocation. These are measured by the total time a household spends on agricultural production and the total time spent on non-agricultural employment, respectively. Considering that the total household labor time is a continuous variable, we use Ordinary Least Squares (OLS) to empirically analyze the impact of mechanization-driven farmland consolidation on total household agricultural labor time and total non-agricultural labor time. The specific model settings are as follows:

$$Labor\_farmtime_{i}=\alpha_{0}+\alpha_{1}High\_sd_{i}+\alpha_{2}Control_{i}+\varepsilon_{i}$$ (3)

$$Labor\_nofarmtime_{i}=\beta_{0}+\beta_{1}High\_sd_{i}+\beta_{2}Control_{i}+\varepsilon_{i}$$ (4)

Where Labor_farm_i_ and nofarm_i_ represent the total duration (in months) of agricultural labor input and non-agricultural labor input of the i-th farmer, respectively, and the remaining parameter settings are the same as in formulas (1) and (2).

(3) Moderating effect model. Based on the baseline regression, interaction terms of mechanization-driven farmland consolidation × agricultural machinery purchase and mechanization-driven farmland consolidation × agricultural machinery service purchase are introduced for regression estimation. The model is set up as follows:

$$Labor\_farm_{i}=\alpha_{0}+\alpha_{1}High\_sd_{i}+\alpha_{2}mach\_buy_{i}+\alpha_{3}High\_sd_{i}\times mach\_buy_{i}+\alpha_{4}Control_{i}+\varepsilon_{i}$$ (5)

$$Labor\_farm_{i}=\alpha_{0}+\beta_{1}High\_sd_{i}+\beta_{2}mach\_servce_{i}+\beta_{3}High\_sd_{i}\times mach\_servce_{i}+\beta_{4}Control_{i}+\varepsilon_{i}$$ (6)

Wherein, mach_buy_i_ and mach_servce_i_ represent agricultural machinery purchase (0–1) and agricultural machinery service purchase (0–1), respectively; High_sd_i_ × mach_buy_i_ and High_sd_i_ × mach_servce_i_ are the interaction terms of mechanization-driven farmland consolidation × agricultural machinery purchase and mechanization-driven farmland consolidation × agricultural machinery service purchase, used to examine whether mechanization-driven farmland can influence farmers’ agricultural labor input by accelerating the “machinery-labor” substitution process; Control_i_ is the control variable; α and β are the coefficients to be estimated; ε represents the random error term; and the meaning of other parameter settings is the same as in formulas (1) and (2).

The interaction terms are used to test the theoretical mechanism. After high-standard farmland improves operating conditions, they may, on the one hand, strengthen the capital-deepening pathway of “machinery purchase → specialized operations,” thereby increasing inputs related to labor organization and equipment maintenance in agriculture; on the other hand, they may reinforce the “service outsourcing → labor substitution” pathway, further reducing the demand for household agricultural labor. If the coefficient on the former interaction term is positive and that on the latter is negative, the results are consistent with the theoretical expectations, thereby providing stronger empirical support for the analytical framework.

All models identify the average treatment effect conditional on controlling for individual- and village-level observable characteristics and regional fixed effects, and they conduct robustness checks—via variable substitutions, instrumental variables, and PSM—to mitigate concerns about endogeneity and selection bias.

### Data source

All investigation procedures in this study followed relevant ethical guidelines. Due to the fact that the research data is anonymous and does not involve any sensitive personal information or medical data, this study has obtained exemption approval from Yantai Agricultural Science Research Institute in Shandong Province. In the process of data collection, we still ensured the principle of informed consent: the researcher orally read out an informed consent statement to the respondents before the start of each questionnaire, which includes the research purpose, data use, anonymous processing, and voluntary participation. If the interviewee agrees to participate, start filling out the questionnaire; If you do not agree, terminate the access. The survey respondents did not include minors.

The empirical analysis data for this study comes from a survey of 630 grain-farming households conducted by the research team in Shandong Province from November 2024 to March 2025. S1 Table contains the dataset underlying this study, which includes data on demographics, farm management, and mechanization behaviors of the surveyed laborers. The questionnaire included questions on: agricultural labor time (months/person), area of farmland transferred in/out (ha), application rates of chemical fertilizer/organic fertilizer (kilograms/ha), and agricultural machinery service expenditure (RMB/ha), as well as the age and education level of the household head, the number of family laborers, the degree of land fragmentation (number of plots/total area), and village topographic characteristics. In addition, we integrated data from the Shandong Statistical Yearbook (2005–2024) and consulted with the farmland construction management departments of the agricultural and rural affairs bureaus in various prefecture-level cities to obtain indicators such as the cumulative construction area of mechanized farmland. Table 2 shows the grain production and mechanized farmland improvement situation in Shandong Province in 2024.

**Table 2:** Grain Production Status and the Development of Mechanization-driven Farmland.

	Index	Numerical values
**Grain Production**	Gross agricultural output value (100 million RMB)	5659.00
	Total grain output (10,000 tons)	5710.20
	Wheat	2716.56
	Maize MAize	2589.52
	Grain Sown Area (Thousand Hectares)	8412.60
	Wheat	4024.20
	Maize MAize	3897.00
**Mechanization-driven Farmland**	Construction Area (500 ha)	519.66
	Construction Ratio (%)	63.40

Data source: 2024 Shandong Statistical Yearbook, publicly available reports from the Shandong Provincial Department of Industry and Information Technology, and the Department of Agriculture and Rural Affairs.

## Empirical results analysis

### Analysis of baseline regression results

**Fig 3:**
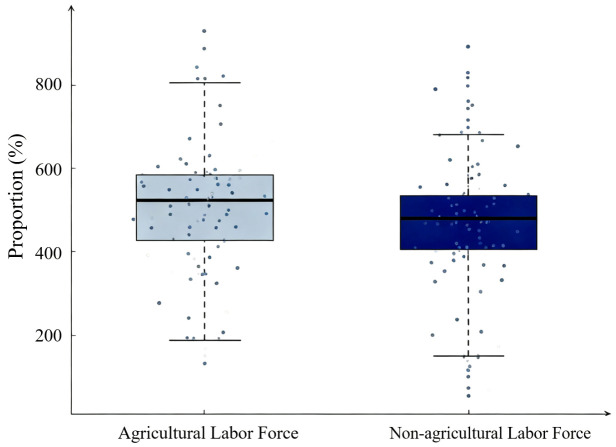
Distribution of agricultural and non-agricultural labor shares among households (%).

As can be seen from Table 3, Pseudo R^2^ increased markedly after adding key explanatory and control variables, indicating a good degree of fit. The empirical results show that mechanized farmland construction significantly reduces the proportion of farm household agricultural labor by approximately 8.4 percentage points at the 1% statistical level, verifying Hypothesis 1. Agricultural machinery purchase significantly increases the proportion of agricultural labor by 5.3% at the 5% statistical level, reflecting an increase in farmers’ enthusiasm for agricultural production. Conversely, the purchase of agricultural machinery services significantly reduces the proportion of agricultural labor by 7.5 percentage points at the 5% statistical level. This indicates that there are significant differences in the impact of different mechanization methods on labor factor allocation: machinery purchase promotes specialized agricultural operations and increases agricultural input, while service purchase alleviates labor constraints and reduces agricultural input. Fig 3 presents the distributions of agricultural and non-agricultural labor shares across households, highlighting substantial cross-household heterogeneity in labor allocation that aligns with the regression-based reallocation effects.

**Table 3:** Estimation Results of the Impact of Mechanization-driven Farmland Consolidation on Farmers’ Labor Allocation.

Variables	Agricultural Labor Allocation Ratio		Non-agricultural Labor Allocation Ratio	
	(1)	(2)	(3)	(4)
	Tobit			
**Mechanization-driven Farmland Consolidation**	–0.056^**^ (0.016)	–0.085^***^ (0.019)	0.042 (0.019)	0.027 (0.019)
**Agricultural Machinery Purchase**		0.047^**^ (0.019)		–0.002 (0.016)
**Agricultural Machinery Service Purchase**		–0.067^**^ (0.024)		0.057^**^ (0.031)
**Decision-maker Gender**		0.001 (0.031)		–0.035 (0.034)
**Decision-maker Age**		0.003^***^ (0.001)		–0.001 (0.001)
**Decision-makers’ Years of Education**		–0.004^*^ (0.003)		0.005^**^ (0.004)
**Decision-maker’s Health Status**		–0.004 (0.007)		0.003 (0.004)
**Agricultural Technology Training for Policymakers**		–0.022 (0.021)		0.012 (0.017)
**Household Size**		–0.016^***^ (0.005)		0.019^***^ (0.006)
**Aging Index**		0.064^**^ (0.026)		–0.049 (0.039)
**Farmland Operation Scale**		0.027^**^ (0.016)		–0.001 (0.015)
**Squared Term of Farmland Operation Scale**		–0.001 (0.004)		–0.002 (0.005)
**Number of Plots**		0.005^***^ (0.002)		–0.005^**^ (0.002)
**Farmland Quality**		0.030^***^ (0.002)		–0.005^**^ (0.002)
**Household Gross Income**		–0.049^***^ (0.014)		0.052^***^ (0.016)
**Agricultural Income Share**		0.165^***^ (0.040)		–0.269^***^ (0.051)
**Farmland Consolidation Experience**		0.019 (0.017)		–0.015 (0.018)
**Agricultural Production Subsidies**		0.003 (0.003)		–0.003 (0.003)
**The Distance of the Village from the Township Government**		–0.010^***^ (0.003)		0.010^***^ (0.003)
**Village Transportation**		–0.115^***^ (0.049)		0.097^*^ (0.036)
**Village Secondary and Tertiary Industries**		–0.021 (0.019)		–0.008 (0.017)
**Per Capita Farmland Area in the Village**		0.021^***^ (0.006)		–0.013^**^ (0.006)
**Is the Village Located in A Plain?**		0.030 (0.026)		–0.014 (0.026)
**Regional Dummies**	Uncontrolled	Control	Uncontrolled	Uncontrolled
**Constant Term**	0.531^***^ (0.012)	0.802^***^ (0.221)	0.230^***^ (0.026)	–0.417 (0.245)
**Sample Size**	630	630	630	630
**Pseudo R** ^**2**^	0.004	0.396	0.002	0.365

Note: *, **, and *** represent significance levels of 10%, 5%, and 1%, respectively. Values in parentheses are robust standard errors, and this convention applies throughout. The results reported in the table are marginal effects.

**Fig 4:**
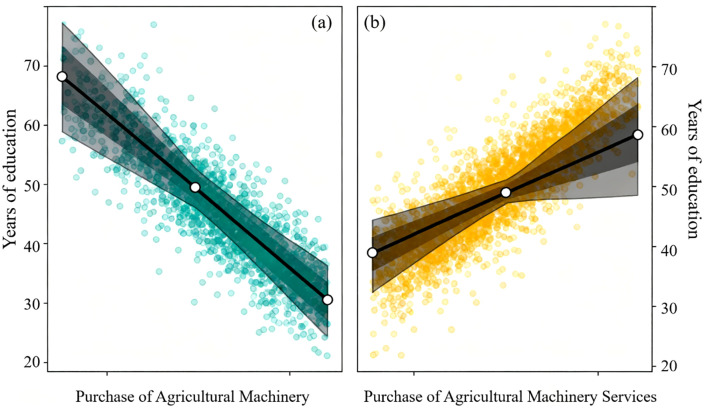
Cultivated land management scale (ha) by machinery purchase and service purchase status.

**Fig 5:**
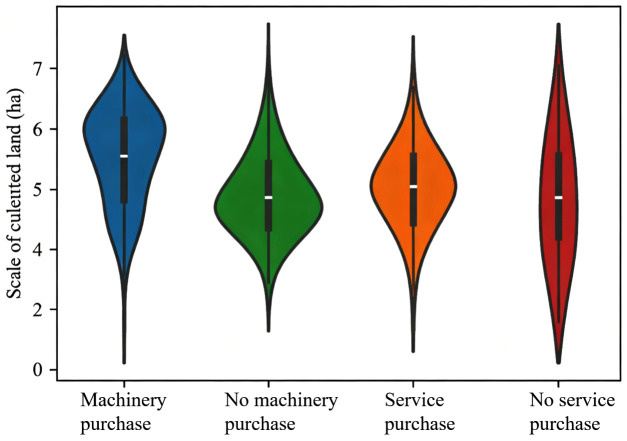
Years of education by agricultural machinery and service adoption status.

Column (3) of Table 3 shows that the estimation results for the control variables are consistent with expectations. The characteristics of farm household decision-makers, household characteristics, and village characteristics all have a significant impact on the proportion of agricultural labor. An increase in the decision-maker’s age increases agricultural labor input, while an increase in education level reduces it. Fig 4 shows the distribution of educational years by machine and service adoption status, divided by population mean and 95% confidence interval. This demonstrates the systematic differences in education between adopters and non-adopters, consistent with the pattern described above. An increase in the household population increases the proportion of non-agricultural labor, while an increase in the degree of aging increases agricultural labor input. The farm land management scale has an inverted U-shaped relationship with agricultural labor. Fig 5 shows the distribution of agricultural land management scale among farmers based on their purchase of machinery and services. The average cultivated land area managed by households that purchase agricultural machinery is 2.17 hectares, which is 47.62% higher than the average cultivated land area of households that do not purchase agricultural machinery. In contrast, the average land area managed by households purchasing agricultural machinery services is 13.98% lower than that of non-service buyers. An increase in the number of plots increases agricultural labor demand, an improvement in farmland quality increases enthusiasm for farming. Household total income has a significant negative impact on the proportion of agricultural labor, while an increase in the proportion of agricultural income increases agricultural labor input. The further the village is from the township government and the improvement of transportation conditions both reduce the proportion of agricultural labor. An increase in per capita cultivated land area in the village promotes large-scale agricultural operations and increases the proportion of agricultural labor.

### Robustness checks and endogeneity discussion

(1) Replacing Variables. The robustness of the results is tested by replacing the core explanatory variable (proportion of farmland construction driven by mechanization) and the explained variable (changing from relative proportion to absolute indicator of total labor input). The results show that the impact of mechanized farmland construction on farmers’ agricultural labor input remains significantly negative, while the impact on non-agricultural labor input is not significant. This confirms that mechanized farmland construction indeed reduces farmers’ agricultural labor input, and the baseline regression results are robust and reliable. The results are shown in Table 4.

**Table 4:** Estimation Results of Robustness Checks: Alternative Variables.

Variables	Agricultural Labor Allocation Ratio	Non- agricultural Labor Allocation Ratio	Agricultural Labor Hours	Non-agricultural Labor Hours
	Tobit	Tobit	OLS	OLS
**Proportion of Mechanization-driven Farmland Consolidation**	–0.059^***^ (0.027)	0.022 (0.017)		
**Mechanization-driven Farmland Development**			–1.145^**^ (0.462)	0.490 (0.695)
**Agricultural Machinery Purchase**	0.049^*^ (0.026)	–0.003 (0.027)	0.265 (0.514)	–0.877 (0.632)
**Agricultural Machinery Service Purchase**	–0.063^**^ (0.024)	0.054^**^ (0.018)	–1.245^**^ (0.702)	1.365^*^ (0.752)
**Control Variables**	Control	Control	Control	Control
**Regional Dummy Variables**	Control	Control	Control	Control
**Constant Term**	0.795^***^ (0.221)	–0.369^*^ (0.295)	13.747^***^ (3.845)	–24.156^***^ (4.265)
**Sample Size** **Adj·R** ^**2**^ **/Pseudo R** ^**2**^	630 0.365	630 0.315	630 0.196	630 0.485

Note: *, **, and *** represent significance levels of 10%, 5%, and 1%, respectively. Values in parentheses are robust standard errors, and this convention applies throughout. The results reported in the table are marginal effects.

(2) Considering Potential Endogeneity Issues. Although mechanized farmland construction can be regarded as an exogenous variable, its implementation process may be related to factors such as local economic development level and topographic features, and it requires the consent of the majority of farmers in the project area, which may lead to reverse causality. To solve this problem, the instrumental variable method is used, and “the mechanized farmland construction of other farmers in the same township except for the household” is selected as the instrumental variable, which meets the requirements of correlation and exogeneity. The results are shown in Table 5. The IV-Tobit estimation results show that the impact of mechanized farmland construction on the proportion of agricultural labor is significantly negative at the 1% statistical level, and the impact on the proportion of non-agricultural labor is still not significant, which is consistent with the baseline regression results, and once again verifies hypothesis 1.

**Table 5:** Addressing potential endogeneity: IV-Tobit model estimation results.

Variables	Agricultural Labor Allocation Ratio	Non-agricultural Labor Allocation Ratio
	IV-Tobit	IV-Tobit
**Mechanization-driven Farmland Development**	–0.132** (0.050)	0.080 (0.048)
**Agricultural Machinery Purchase**	0.069** (0.036)	–0.014 (0.031)
**Agricultural Machinery Service Purchase**	–0.064** (0.029)	0.063** (0.024)
**Controlled Variables**	Control	Control
**Regional Dummy Variables**	Control	Control
**Constant Term**	0.745** (0.299)	–0.312 (0.301)
**Instrumental Variable Coefficient Estimation**		0.965*** (0.036)
**F-value**		48.612
**Sample Size**	630	630

Note: * and **represent significance levels of 10% and 5%, respectively. Values in parentheses are robust standard errors, and this convention applies throughout. The results reported in the table are marginal effects.

(3) Propensity Score Matching (PSM). Considering that the implementation of mechanized farmland construction projects may be affected by factors such as regional economic development, geomorphological type, farmland scale, and village trust, resulting in non-randomness, which may lead to selection bias. As shown in Table 6, after re-estimating using the PSM method, it is found that the average treatment effect of mechanized farmland construction on the proportion of agricultural labor calculated based on the four matching methods is significantly negative, with an average value of −0.083; while the average treatment effect on the proportion of non-agricultural labor is positive, but did not pass the significance test. This further confirms the robustness of the research conclusions: mechanized farmland construction does reduce farmers’ agricultural labor input, but has no significant impact on non-agricultural labor input.

**Table 6:** Estimated Average Treatment Effect on the Treated (ATT) Based on Four Matching Methods.

Matching Methods	Proportion of Agricultural Labor Force			Proportion of Non-Agricultural Workforce		
	ATT	Standard error	T-value	ATT	Standard error	T-value
**Kernel Matching**	–0.079*	0.036	1.874	0.051	0.043	1.025
**Nearest Neighbor Matching**	0.098**	0.043	2.279	0.068	0.044	1.362
**Caliper Match**	0.079**	0.040	1.595	0.036	0.042	0.997
**Locally Linear Regression Fitting**	0.036**	0.037	2.054	0.037	0.053	0.526
**Average**	–0.078			0.051		

Note: * and **represent significance levels of 10% and 5%, respectively.

### Mechanism verification

(1) Mechanization-driven farmland construction, agricultural machinery purchase, and agricultural labor input. As shown in Table 7(1), the coefficient of the interaction term between mechanization-driven farmland construction and agricultural machinery purchase is 0.185, which is significant at the 1% statistical level. This indicates that mechanization-driven farmland construction strengthens the promotion effect of agricultural machinery purchase on the proportion of agricultural labor input by farmers. This implies that mechanization-driven farmland construction promotes agricultural machinery purchase, which in turn promotes specialized agricultural operations, leading to an increase in agricultural labor input. This result suggests that farmers purchase agricultural machinery primarily to engage in large-scale or specialized operations and tend to invest family resources in agricultural production to maximize returns.
(2) Mechanization-driven farmland construction, agricultural machinery service purchase, and agricultural labor input. Table 7(2) shows that the impact of the interaction term between mechanization-driven farmland construction and agricultural machinery service purchase on the proportion of agricultural labor is significantly negative at the 1% statistical level. This indicates that mechanization-driven farmland construction strengthens the reducing effect of agricultural machinery service purchase on agricultural labor input by farmers, increased farmers’ purchase of agricultural machinery services, alleviating household labor constraints, thereby reducing agricultural labor input. In summary, mechanization-driven farmland construction improves conditions for mechanized farming, accelerating the “machinery-labor” substitution process, but the impact of different mechanization implementation methods differs: machinery purchase promotes specialized agricultural operations and increases labor input, while service purchase alleviates labor constraints and reduces input, verifying Hypothesis 2.

**Table 7:** Results of Mechanism Tests on the Impact of Mechanization-driven Farmland Consolidation on Agricultural Labor Input.

Variables	Proportion of Agricultural Labor Force	
	(1)	(2)
**Mechanization-driven Farmland Consolidation**	–0.067^**^ (0.019)	–0.072^***^ (0.017)
**Agricultural Machinery Purchase**	0.026 (0.024)	0.036 (0.016)
**Agricultural Machinery Service Purchase**	–0.049^**^ (0.029)	–0.061^**^ (0.021)
**Mechanization-driven Farmland Consolidation × Agricultural Machinery Purchase**	0.176^**^ (0.037)	
**Mechanization-driven Farmland Consolidation × Agricultural Machinery Service Purchase**		–0.148^***^ (0.036)
**Controlled Variables**	Control	Control
**Regional Dummy Variables**	Control	Control
**Constant Term**	0.169^**^ (0.196)	0.785^**^ (0.196)
**Sample Size** **Pseudo R** ^**2**^	630 0.386	630 0.409

Note: *, **, and *** represent significance levels of 10%, 5%, and 1%, respectively. Values in parentheses are robust standard errors, and this convention applies throughout. The results reported in the table are marginal effects.

### Heterogeneity analysis

Table 8 reveals significant heterogeneity in the impact of mechanized farmland construction on household labor allocation across different types of farm households. Regarding aging, households with elderly members (aged 65 and above) are more significantly affected by mechanized farmland construction, experiencing a reduction of approximately 17.1 percentage points in their agricultural labor force share, which is 12.6% higher than non-elderly households. This may be due to the declining physical capacity of elderly farmers, with mechanized farmland construction significantly alleviating their farming burden by promoting the substitution of machinery for labor. In terms of farm size, mechanized farmland construction significantly reduces the agricultural labor force share of small-scale farmers (≤1 ha), while having no significant impact on large-scale farmers. This is because small-scale farmers are often part-time farmers, and farmland construction allows them to free up labor from agriculture for non-agricultural employment, whereas large-scale farmers are more inclined to maintain specialized agricultural operations. Households with agricultural income accounting for less than 30% of their total income are more significantly affected by mechanized farmland construction, experiencing an average reduction of approximately 10.0 percentage points in their agricultural labor force share, while there is no significant impact on households with a high proportion of agricultural income. This indicates that the proportion of agricultural income reflects the importance of agriculture in household livelihoods, and high-proportion households tend to maintain resources in the agricultural sector. In terms of topography, farm households in non-plain areas are more significantly affected, with an average reduction of approximately 14.7% in their agricultural labor force share, which is 6.6 percentage points higher than in plain areas. This is attributed to the originally poor site conditions and severe fragmentation of plots in non-plain areas. Mechanized farmland construction significantly improves their mechanization conditions through land leveling and road construction, making the labor reduction effect more pronounced.

**Table 8:** Estimated Impact of Mechanization-driven Farmland Consolidation on Agricultural Labor Input of Farmers with Different Endowments.

Sample Grouping		Proportion of Agricultural Labor Force	Sample size	Pseudo R^2^
**Aging Level Grouping**	No seniors (65+)	–0.034 (0.031)	416	0.415
	Seniors (65+)	–0.165*** (0.050)	314	0.841
**Farm Size Categories**	< 1 ha	–0.083*** (0.029)	390	0.504
	≥ 1 ha	–0.065 (0.052)	129	0.877
**Agricultural Income Share Groups**	<Mean (30%)	–0.100** (0.038)	368	0.299
	≥ Mean (30%)	–0.062 (0.037)	186	0.635
**Village Terrain Grouping**	Plain Areas	–0.069** (0.036)	309	0.495
	Non-plain Areas	–0.241*** (0.070)	296	0.506

Note: *, **, and *** represent significance levels of 10%, 5%, and 1%, respectively. Values in parentheses are robust standard errors, and this convention applies throughout. The results reported in the table are marginal effects.

## Discussion

Based on research data and the Chinese context of agricultural modernization, the findings of this study provide a novel policy perspective for understanding the multifaceted impacts of mechanization-driven farmland consolidation. Research had shown that Shandong Province had achieved an 8.4% reduction in agricultural labor through mechanized farmland construction, fundamentally revealing the differentiated mechanisms between mechanized services and purchasing traditional agricultural machinery. Unlike the state-owned machinery rental model in the Red River Delta of Vietnam [39] or the large-scale farm subsidy policies in Portugal [40], China’s farmer-led “gradual substitution” model, where 84.1% of farmers choose socialized services rather than purchasing agricultural machinery, effectively alleviates the dual constraints of land fragmentation (an average of 5.2 plots per household) and aging (an average age of 55.6 years). This model is particularly prominent among farmers in mountainous areas [41], where the labor release effect is 47% higher than in plain areas, indicating an urgent need to develop agricultural machinery adapted to the terrain.

The policy paradox revealed by the study warrants attention: While improving efficiency, improvements in farmland construction infrastructure may accelerate the intergenerational rupture in rural occupations. The agricultural labor input of elderly farmers (over 65 years old, accounting for 28.4%) decreased by 17.1%, far exceeding that of non-elderly groups, necessitating the inclusion of old-age security and skill conversion mechanisms in policy design. Shandong Province could pilot a combination of mechanization-driven farmland subsidies and training programs for elderly agricultural machinery operators, transforming the released labor force into a professional supply for the agricultural service sector (Table 9). For farmland operators with an operating scale exceeding 0.67 hectares, the study found that the efficiency improvement did not meet expectations, possibly due to the lag in post-production processing facilities, leading to limited value chain enhancement, indicating that the next stage of policy needs to extend from the production end to the back end of the value chain.

**Table 9:** Policy Implementation Roadmap.

Policy Stage	Key Action	Target Group	Timeline
**Phase 1**	Machinery leasing subsidies	Smallholders (<5 ha)	2026-2028
**Phase 2**	Elderly operator training	Farmers >65 years	2027-2030
**Phase 3**	Digital integration	Service providers	2029-2035

On this basis, we further explore labor allocation and substitution potential. We compare two sets of indicators reported in Tables 7 and 8: the share of non-agricultural labor allocation alongside the proportion of nonfarm working hours, and the share of agricultural labor allocation alongside the proportion of on-farm working hours. Given the observed allocation structure in our sample, we discuss the feasible scope for redirecting a portion of nonfarm labor to mechanized field operations. This potential reallocation is shaped by household-level health constraints, learning costs and skill thresholds, as well as participation in nonfarm roles that support input procurement and produce marketing [42,43]. Because these supporting activities are typically seasonal—aligned with planting and harvesting periods [44]—a comparative assessment of substitution effects can help identify the relative comparative advantages of nonfarm versus on-farm labor and reveal key household-level frictions that constrain mobility, thereby clarifying the boundaries and constraints of labor reallocation under mechanization [45].

We also find substantial scope to strengthen technology uptake through targeted training for production decision-makers. The sample indicates a median of 7 years of formal schooling among decision-makers (Table 1), providing a foundation for aligning training content with learners’ educational capacity. Prior studies have documented that more educated farmers are better positioned to understand and adopt mechanization, digital tools, and modern agronomic practices [46,47]. Accordingly, agricultural extension services should be reinforced and better tailored to existing educational levels where systems are already established. Where extension networks are weak or unevenly implemented, increased targeted investment and institutional support could improve knowledge transfer and the diffusion of on-farm innovations.

Survey evidence indicates that average operational scales remain small and that there is room to increase machinery ownership. This profile exacerbates the challenges smallholders face in mechanization investment and land-use efficiency. Prior research shows that, under limited per capita arable land, cooperative arrangements or outsourced machinery services can improve access to equipment and enable a more intensive allocation of land and machinery resources [48,49]. It is therefore advisable to explore cooperative joint operations or to promote private-sector machinery rental services to support rational land consolidation and mechanization upgrading among staple crop producers, while leveraging existing rural infrastructure and service availability.

In terms of international experience, China’s practice provides a new paradigm for regions with global land fragmentation. Although Shandong Province’s land fragmentation index (5.2) is higher than the national level of India (3.8), the scale-neutral characteristics of mechanization services have successfully overcome the limitations of traditional economies of scale [50]. This contrasts sharply with the collective ownership reforms in sub-Saharan Africa, which have suffered from insufficient investment incentives due to unclear property rights [51]. However, the credit bundling mechanism of Vietnamese cooperatives inspires us to further reduce the participation costs of marginal farmers through innovative tools such as “agricultural machinery service vouchers + microcredit” [52].

Despite the consistency of our results across multiple robustness checks [53], we acknowledge several limitations. First, on the data and measurement side, survey responses may be subject to recall and social desirability biases; key variables such as labor input and mechanization services are also unlikely to be entirely free from measurement error and selective response, which may introduce noise into the estimates. Second, in terms of identification, although we control for rich individual-, household-, and village-level characteristics and implement robustness checks—including alternative variable definitions, instrumental variables, and PSM—potential unobserved heterogeneity and endogeneity cannot be completely ruled out. Finally, with respect to external validity, the samples geographic coverage and observation window are limited; differences in policy environments and factor endowments across regions may affect the applicability of the conclusions, so extrapolation to other regions or periods should be undertaken with caution.

Regarding future research prospects, the study aims to break through in three frontier areas: First, research needs to track the long-term human capital effects of labor saving. Current data have not revealed whether the saved farming time is transformed into non-agricultural skill accumulation. Second, in terms of digital integration, there is a huge gap between Shandong Province’s mechanization rate of 86.5% and the smart agriculture penetration rate of 23%. It is recommended to break down the “digital access barriers” for small farmers through policies such as rental subsidies for IoT equipment. Finally, ecological cost accounting should be incorporated into the policy evaluation framework. Data show that fertilizer input in mechanized plots increased by 8.4%, which requires incorporating soil carbon sink monitoring into the acceptance standards for mechanization-driven farmland, establishing a “productivity-ecological security” dual-track evaluation system. These findings provide a theoretically innovative and policy-feasible Chinese solution for global agricultural transformation.

## Conclusion

Mechanization-driven farmland construction significantly reduces agricultural labor input while exhibiting no clear impact on non-agricultural labor. This outcome arises mainly from improved conditions for mechanized operations, which accelerate the substitution of labor with machinery. The effect, however, varies by mechanization mode: while purchasing machinery may increase agricultural labor input, opting for machinery services tends to reduce it. The policy exerts more pronounced effects on older farmers, small-scale operators, those with lower shares of agricultural income, and farmers in non-plain areas. Further analysis reveals that farmland construction primarily reduces agricultural labor input among members aged 45 and above and promotes non-agricultural employment for those between 45 and 65. However, due to the limited off-farm employability of older laborers, the overall effect on non-agricultural labor remains insignificant.

Although this study identifies causal relationships and heterogeneous pathways in labor allocation resulting from mechanization-driven farmland consolidation, certain limitations remain. Future research should focus on: (1) Carry out a long-term follow-up study to evaluate the dynamic effect and sustainability of mechanization driven farmland integration. (2) Expand the research scope and compare the policy effect differences in different regions and different crop types. (3) In depth study on the quality of non-agricultural employment and the effect of human capital accumulation of the released labor force. Addressing these aspects would not only mitigate the constraints of cross-sectional data but also offer a more robust empirical basis and policy guidance for agricultural modernization in China.

## Supporting information

S1 Table Supporting Information. (XLSX)
